# Hydrogen sulfide ameliorates cognitive dysfunction in streptozotocin-induced diabetic rats: involving suppression in hippocampal endoplasmic reticulum stress

**DOI:** 10.18632/oncotarget.19448

**Published:** 2017-07-22

**Authors:** Wei Zou, Juan Yuan, Zhuo-Jun Tang, Hai-Jun Wei, Wei-Wen Zhu, Ping Zhang, Hong-Feng Gu, Chun-Yan Wang, Xiao-Qing Tang

**Affiliations:** ^1^ Department of Neurology, Nanhua Affiliated Hospital, University of South China, Hengyang 421001, Hunan, P.R. China; ^2^ Institute of Neuroscience, Medical College, University of South China, Hengyang 421001, Hunan, P.R. China; ^3^ Department of Pathophysiology, Medical College, University of South China, Hengyang 421001, Hunan, P.R. China; ^4^ Hunan Province Cooperative Innovation Center for Molecular Target New Drug Study, Hengyang 421001, Hunan, P.R. China

**Keywords:** cognitive dysfunction, diabetes, endoplasmic reticulum stress, hydrogen sulfide, streptozotocin

## Abstract

Diabetes induces impairment in cognitive function. There is substantial evidence that hippocampal endoplasmic reticulum (ER) stress is involved in diabetic cognitive impairment. Hydrogen sulfide (H_2_S) attenuates the learning and memory decline in experimental Alzheimer's disease and inhibits the hippocampal ER stress in homocysteine-exposed rats. Therefore, this aim of the present work was to investigate whether H_2_S ameliorates the diabetic cognitive dysfunction involving inhibition of hippocampal ER stress. In the present work, we found that stretozotocin (STZ, 40 mg/kg)-induced diabetic rats exhibited impairment in cognitive function, as judged by the novel objective recognition task (NOR) test, the Y-maze test and the Morris water maze (MWM) test. Notably, treatment of diabetic rats with sodium hydrosulfide (NaHS, a donor of H_2_S, 30 or 100 μmol/kg/d, for 30 d) significantly reversed diabetes-induced impairment in cognitive function. We also found that STZ (40 mg/kg)-induced diabetic rats exhibited hippocampal ER stress, as evidenced by upregulations of glucose regulated protein 78 (GRP78), C/EBP homologous protein (CHOP), and cleaved caspase-12 in the hippocampus. However, treatment with NaHS (30 or 100 μmol/kg/d, for 30 d) markedly suppressed the increases in GRP78, CHOP, and cleaved caspase-12 expressions in the hippocampus of diabetic rats. In addition, we noted that NaHS (30 or 100 μmol/kg/d, for 30 d) significantly enhanced the generation of hippocampal endogenous H_2_S in STZ-induced diabetic rats. These results suggest that H_2_S exhibits therapeutic potential for diabetes-associated cognitive dysfunction, which is most likely related to its protective effects against hippocampal ER stress.

## INTRODUCTION

Diabetes mellitus (DM), the most common endocrine disorder disease inducing cognitive impairment [1, 2], is increasing at an alarming rate and has been becoming a major public health concern [3]. Diabetics exhibit middle impairments in cognitive function and have a high risk of affective disorders, dementia and Alzheimer disease (AD) [4, 5]. Many evidence have demonstrated that the cognitive impairments is present in 30% to 40% of elderly DM patients, and the severity of cognitive impairment has a direct relationship with poor glycemic control in these patients [6]. Increasing evidence in diabetic animal models also demonstrated that diabetes induces cognitive impairment and memory loss [7, 8]. However, no specific approaches are able to prevent cognitive deficits induced by diabetes [9]. It play the pivotal role in development of novel therapeutic strategies to prevent the cognitive deficits in DM.

Hydrogen sulfide (H_2_S), the third gaseous transmitter, exerts a series of biological and physiological effects [10–12]. Recently, a growing number of published literature indicates that H_2_S, acting as a neuromodulator, plays an important role in brain functions [13, 14]. H_2_S is shown to promote the induction of hippocampal long-term potentiation in active synapses, suggesting that H_2_S could improve the learning and memory [15]. It has been demonstrated that H_2_S slows down the progression of experimental AD and attenuates their learning and memory declines [16, 17]. Current evidence shows that the synthesis and circulating level of H_2_S decreases in the obese diabetic mice [18], streptozotocin induced type 1 diabetic mice [19], and type 2 diabetic patients [19]. Several researches have used donation of H_2_S as a latent method to protect against the deterioration of diabetic complications, diabetic nephropathy and cardiomyopathy [20–22], and to improve wound healing in type 2 diabetes [23]. Therefore, we speculated that H_2_S is a potential therapeutic approach for intervention of diabetes-associated cognitive defects. The objective of present researches was evaluating the possible ability of H_2_S to counteract the cognitive defect in streptozotocin (STZ)-exposed diabetic rats and the underlying mechanisms.

The endoplasmic reticulum (ER) is the dominating location for synthesis and maturation of secretory protein. A variety of stimulus can damage ER homeostasis and generate the store of unfolded or misfolded proteins and produced many pathological changes, namely ER stress [24]. The molecular markers of ER stress includes Glucose-regulated protein 78 (GRP78), C/EBP homologous protein (CHOP), and cleaved caspase-12. ER stress-accompanied apoptotic cell has been confirmed in various diseases, including diabetes [25]. Also, a variety of neurodegenerative disorders including AD, Parkinson disease, and cerebral ischemia involve in ER stress-induced apoptosis. [26]. The hippocampus plays a key role in certain types of learning and memory and is a crucial brain region susceptible to stress [27, 28]. Recently, the published article confirmed the hippocampal ER stress is involved in diabetic cognitive impairment [29]. Thus, inhibiting ER stress responsed in hippocampus may provide cognitive protection. In parallel to this, H_2_S inhibits 6-hydroxydopamine (6-OHDA)-induced ER stress in a human neuroblastoma cell line (SH-SY5Y) [30] and cardiomyocytic ER stress in cardiomyocytic injury was demonstrated. [31]. We also recently reported that H_2_S prevents hippocampus from homocysteine-arose ER stress [32]. Therefore, our present work examined whether H_2_S suppresses the hippocampal ER stress in STZ-exposed diabetic rats.

In this experiment, we discovered that H_2_S improved the learning and memory dysfunction of diabetic rats and prevented ER stress in the hippocampus. These findings suggested that this possibility that H_2_S as a novel therapeutic approach for treatment of cognitive impairments in diabetes.

## RESULTS

### NaHS enhances the cognitive function of diabetic rats in novel object recognition test

**Figure 1:**
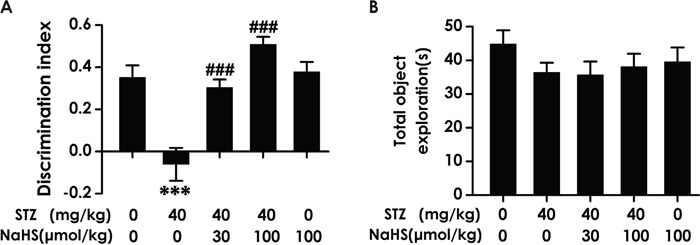
Effect of NaHS on the cognitive decline of STZ-induced diabetic rats in Novel object recognition (NOR) test Rats were once injected with STZ (40 mg/kg) and treated with NaHS (30 and 100 μmol/kg/d, ip) for 30 days. The cognitive performance of rats was test using the NOR task and the discrimination index **(A)** and total object exploration **(B)** were recorded. The data are expressed as mean ± S.E.M. (n= 7–12 per group). *** *P*< 0.001, versus control group; ^###^ *P*< 0.001, versus STZ-treated alone group.

To investigate whether the cognitive function of diabetic rat is impaired and whether H_2_S ameliorates this impairment, we examined the cognitive function of rat using the novel object recognition test. As shown in Figure 1A, the discrimination index in STZ-exposed rats was significantly decreased compared to control. However, NaHS significantly increased the discrimination index of diabetic rats compared to the STZ-treated alone group rats (F4, 31=16.146, P< 0.001). On the other hand, NaHS (100 μmol/kg/d, i.p.) did not affect the discrimination index of no diabetic rats. Furthermore, there was no significant discrepancy of the total exploration time between all groups (F4, 30=0.921, P > 0.05; Figure 1B). These data suggested that NaHS treatment prevents the decline in cognition in diabetic rats.

### NaHS ameliorates the working memory impairment of diabetic rats in Y-maze test

**Figure 2:**
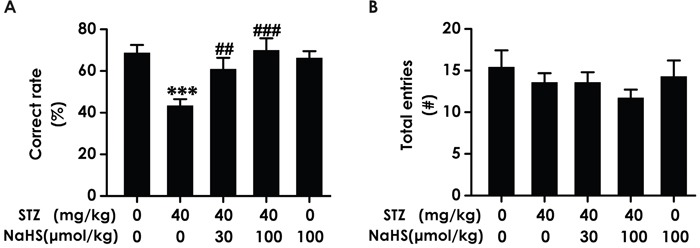
Effect of NaHS on the working memory dysfunction of STZ-induced diabetic rats in Y-maze test Rats were once injected with STZ (40 mg/kg) and treated with NaHS (30 and 100 μmol/kg/d, ip) for 30 days. The cognitive performance of rats was test using the Y-maze task and the correct rate **(A)** and total entries number **(B)** were recorded. The data are expressed as mean ±S.E.M. (n= 7–12 per group). ***P< 0.001, versus control group;^##^*P*< 0.01, ^###^*P*< 0.001, versus STZ-treated alone group.

As an alternative method to confirm H_2_S whether ameliorates working memory dysfunction, we applied the Y-maze test to assess the changes of diabetic rats in working memory functions after treatment with NaHS. Diabetic rats demonstrated an obvious decline in the correct rate compared to blank group; however, treatment of STZ-induced diabetic rats with NaHS inversed the descend in the correct rate compared to those in STZ-treated alone rats (F_4,30_=6.218, P < 0.001; Figure 2A). It should be noted that NaHS (100 μmol/kg/d, i.p.) did not affect the correct rate of rats in control group (Figure 2A). In addition, the total number of entries has no difference in various groups (F_12,22_=0.992, P > 0.05; Figure 2B). These data also indicated that NaHS reverses the working memory dysfunction of diabetic rats.

### NaHS improves spatial learning and memory of diabetic rats in Morris water maze test

**Figure 3:**
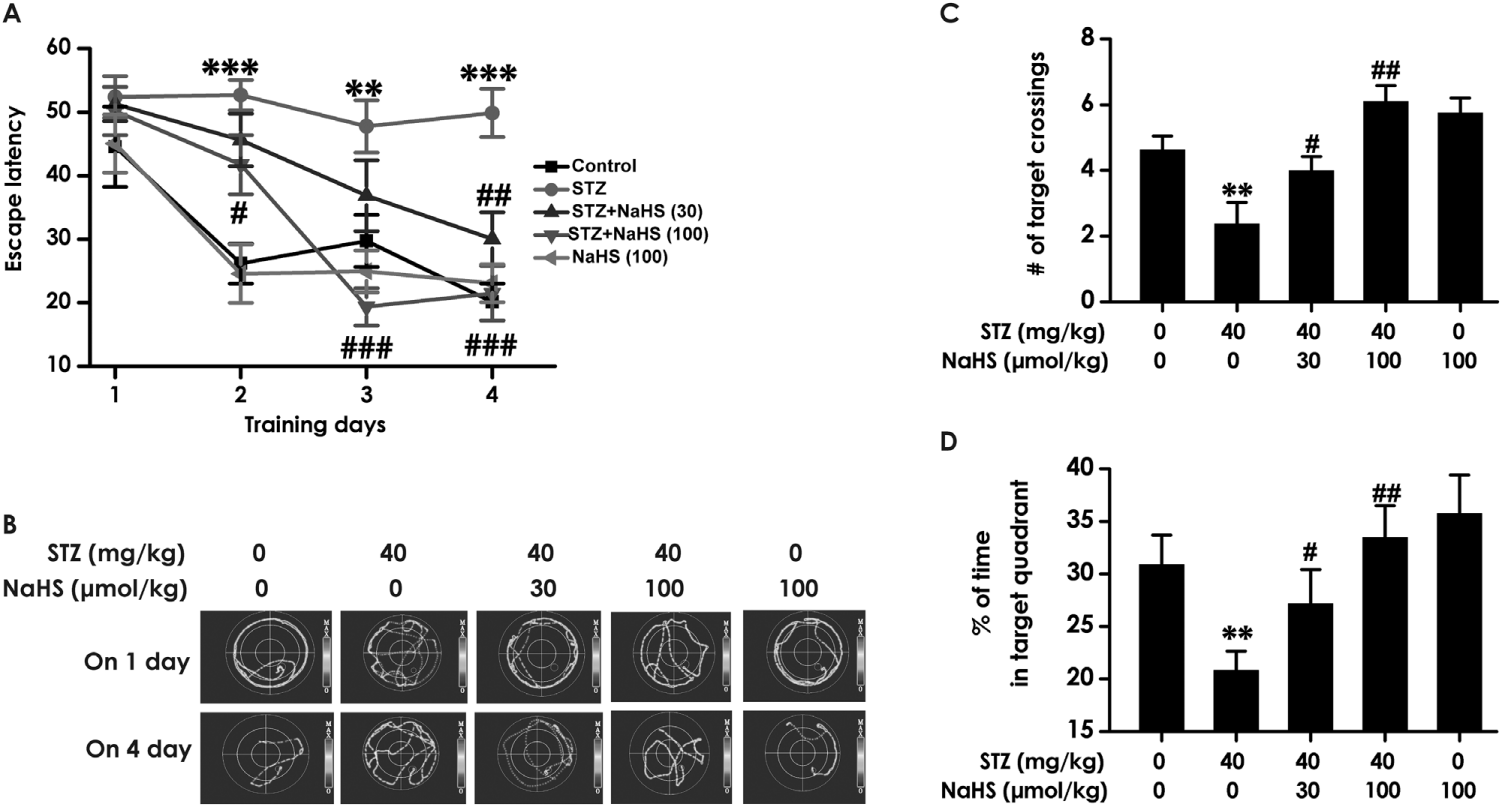
Effects of NaHS on the spatial learning and memory dysfunction of STZ-induced diabetic rats in the acquisition and probe phase of Morris water maze test Rats were once injected with STZ (40 mg/kg) and treated with NaHS (30 and 100 μmol/kg/d, ip) for 30 days. The cognitive performance of rats was test using the Morris water maze task. **(A)** Acquisition profiles: rats were submitted to acquisition of an invisible platform placed in a fixed location (target quadrant) with four swims per day during 4 days. **(B)** Representative swimming tracks of rats searching for the underwater platform at 1^st^ and 4^th^ training day. **(C, D)** One day after finishing the place navigation task (day 5), the platform was removed and the rats were submitted to the probe trial, and the percentage of time spent in the target quadrant **(C)** and the number of times that the animal crossed the target quadrant platform area **(D)** were analyzed. The data are expressed as the mean ± S.E.M. (n=7-12). **P< 0.01, ***p< 0.001, versus control group; ^#^*P*< 0.05, ^##^*P*<0.01, ^###^*P*< 0.001, versus STZ-treated alone group.

To further investigate above-mentioned protective role of H_2_S in cognitive dysfunction of diabetic rats, rats were received the Morris water maze task to measure the spatial learning and memory. As schematized in Figure 3A, all groups over the four training days exhibited a decrease in the escape latency. STZ-induced diabetic rats exhibited significantly higher escape latency on day 2, 3 and 4 during training trials compared with control group rats, suggesting an obvious impairment of spatial learning function. However, treatment with NaHS significantly decreased the escape latency of STZ-induced diabetic rats from training day 2 onward. Figure 3B shows the typical swimming tracks of rats finding the underwater platform. At the 1st training day, the moving distance to search hidden platform had no difference among the five groups. At the 4th training day, diabetic rat exhibited an obvious increase in the distance swam compared with control group; however, a significant decrease emerged on NaHS -treated diabetic group in the distance swam compared to the diabetic group. There were no discrepancy of the escape latency (Figure 3A) and distance swam (F_4, 39_=10.72, P > 0.05; Figure 3B) between control and NaHS (100 μmol/kg/d)-treated alone groups.

In the probe trial, the platform was removed and the rats were placed into the opposite quadrant and allowed to swim freely for 120 s. STZ-induced diabetic rats showed impaired memory, as evidence by their significant decreases in the number of times crossing target quadrant (F_4, 38_=8.65, P < 0.001; Figure 3C) and the duration spent on target quadrant compared with the blank group. (F4, 42=4.4, P < 0.01; Figure 3D). However, NaHS significantly enhanced the duration that the diabetic rats spent on the target quadrant (Figure 3C) and the frequencies that the diabetic rats crossed the target quadrant (Figure 3D) compared to the STZ-treated alone group. NaHS (100 μmol/kg/d, i.p.) alone-treated rats did not show change in this indicators. (Figure 3C, 3D). Taken together, these data suggested that NaHS apparently improved spatial learning and memory that had declined in STZ-induced diabetic rats.

### NaHS improves working memory of diabetic rats in Morris water maze test

**Figure 4:**
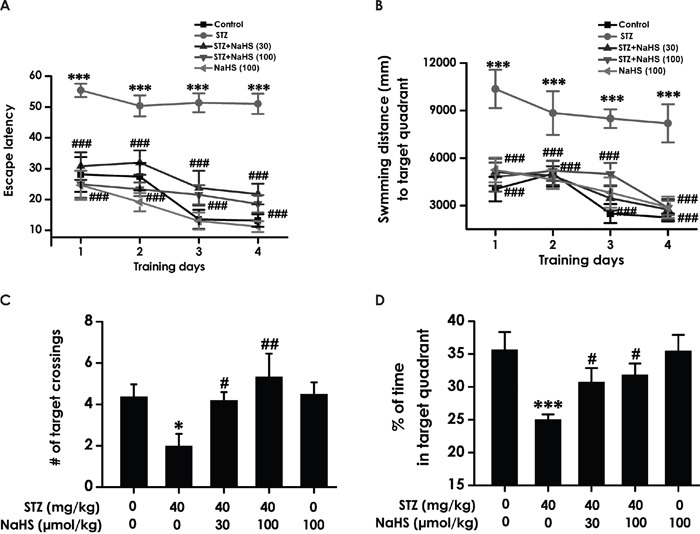
Effect of NaHS on the working memory dysfunction of STZ-induced diabetic rats in the transfer acquisition and probe phases of Morris water maze test **(A, B)** After the visible platform test, the rats were submitted to the transfer acquisition phase of Morris water maze test for four consecutive days, and the latency traveled to find the hidden platform **(A)** and the distance swam in searching for the hidden platform **(B)** in the transfer acquisition phase were recorded. **(C, D)** After the transfer acquisition phase of Morris water maze test, the rats were submitted to the transfer probe phases of Morris water maze test, and the number of times that the animal crossed the target quadrant platform area **(C)** and the percentage of time spent in the target quadrant **(D)** were analyzed. The data are expressed as the mean ± S.E.M. (n=7-12). *P< 0.05, ***P< 0.001, versus control group; ^#^*P*< 0.05, ^##^*P*<0.01, ^###^*P*< 0.001, versus the STZ-treated alone group.

We tested working memory in the transfer phase of the hidden platform version of the water maze. STZ-induced diabetic rats exhibited significant higher escape latency (F4, 40=30.37, P < 0.001; Figure 4A) and a significant enhancement during the swimming distance (F4, 39=10.7, P < 0.001; Figure 4B) in finding the hidden platform on day 1, 2, 3 and 4 during training trials compared to control group rats, implying a significant impairment of working memory process. However, treatment with NaHS significantly decreased the escape latency (Figure 4A) and the swimming distance (Figure 4B) of STZ-induced diabetic rats compared to STZ-treated alone group during 4 d training. There were no differences of the escape latency (Figure 4A) and the swimming distance (Figure 4B) between control and NaHS (100 μmol/kg/d)-treated alone groups. Similarly, in the transfer phase of the probe trial of the water maze, an obvious decreases emerged on STZ-induced diabetic rats in the number of times crossing target quadrant (F4, 35=5.81, P < 0.001; Figure 4C) and the duration spent on target quadrant (F4, 35=4.89, P < 0.01; Figure 4D) However, treatment with NaHS significantly increased the number of times that the diabetic rats crossed the target quadrant (Figure 4C) and the duration that the diabetic rats spent on the target quadrant (Figure 4D) compared to STZ-treated alone group. NaHS (100 μmol/kg/d, i.p.) did not alter the number of times crossing target quadrant (Figure 4C) and the time spent on target quadrant (Figure 4D) when no STZ was applied. Taken together, these data suggested that NaHS apparently improved working memory that had declined in STZ-exposed diabetic rats.

### Rules out the influences by the changes of vision and motor ability on learning and memory in the rats

**Figure 5:**
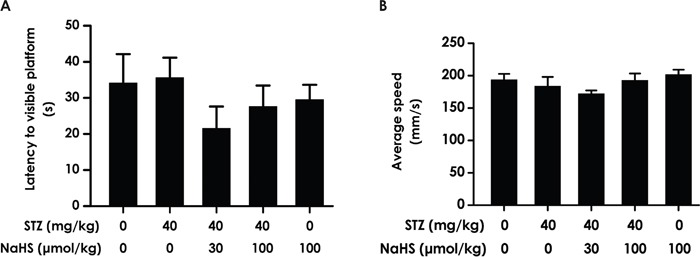
The escape latency and average swimming speed in the visible platform test After the transfer probe test, the rats were submitted to the visible platform test and the latency to reach the platform **(A)** and the average swimming speed of rats **(B)** were recorded.

In order to avert the possibility that the above results are derived from the alterations of vision and motor ability in the rats, we measured the escape latency and the average swimming speed by performing a visible platform test. There was no difference in the escape latencies (F_4, 45_=0.946, P > 0.05; Figure 5A) and swimming speed (F4, 32=0.397, P > 0.05; Figure 5B) among all rats, which indicated the change of all indexes in the these experiments are not due to alterations or difference in visual performance or athletic ability of each groups of rats.

### NaHS downregulats hippocampal GRP78 expression in STZ-induced diabetic rats

**Figure 6:**
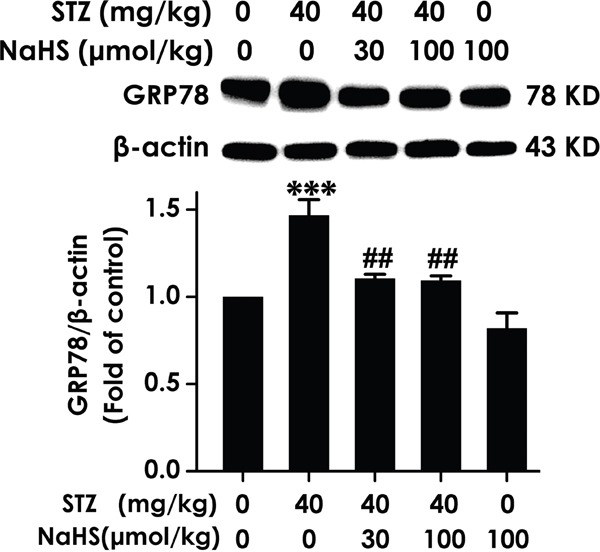
Effect of NaHS on the expression of GRP78 in the hippocampus of STZ-induced diabetic rats Rats were once injected with STZ (40 mg/kg) and treated with NaHS (30 and 100 μmol/kg/d, ip) for 30 days. The expression of GRP78 in the hippocampus of rats was detected by Western blot using anti-GRP78 antibody and β-actin was used as a loading control. Values are expressed as the mean ± S.E.M. (n=3-5). ***P < 0.001, versus control group; ^##^*P* < 0.01, versus STZ-treated alone group.

An important physiological role for hippocampal ER stress in cognitive impairment development was found in diabetes. [29]. This finding had raised the question of whether inhibition of hippocampal ER stress is existed in H_2_S on the improvement of cognitive function in diabetic rats. To investigate this, we quantified multiple parameters, firstly measure the expression of GRP78, which is a pivotal marker of ER stress. As schematized in Figure 6, hippocampal GRP78 expression was significantly increased in diabetic rats; however, treatment with NaHS clearly decreased the protein expression of hippocampal GRP78 in diabetic rats, indicating the response of diabetes to hippocampal ER stress and the suppressive function of NaHS on STZ-induced hippocampal ER stress.

### NaHS represses hippocampal CHOP expression in STZ-induced diabetic rats

**Figure 7:**
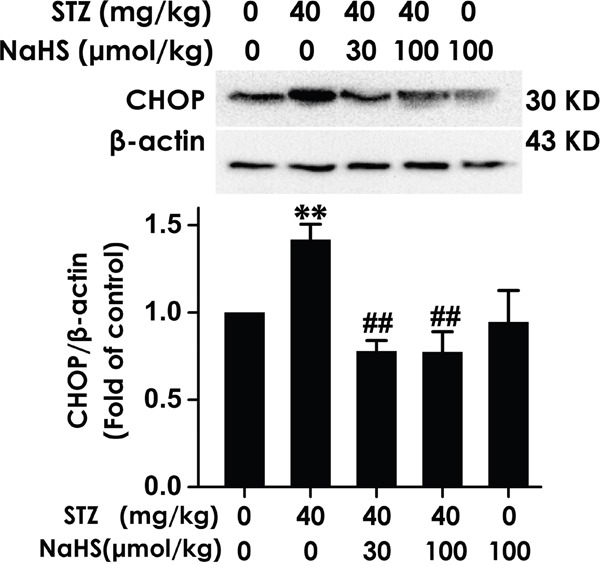
Effect of NaHS on the expression of CHOP in the hippocampus of STZ-induced diabetic rats Rats were once injected with STZ (40 mg/kg) and treated with NaHS (30 and 100 μmol/kg/d, ip) for 30 days. The expression of CHOP in the hippocampus of rats was detected by Western blot using an anti-CHOP antibody and β-actin was used as a loading control. Values are expressed as the mean ± S.E.M. (n=3-5). *P < 0.01, versus control group; ^##^*P* < 0.01, versus STZ-treated alone group.

To further ascertain whether H_2_S inhibits hippocampal ER stress, we provide another result that hippocampal CHOP protein level, which is associated with ER Stress. The expression of this important protein was clearly increased in the hippocampus of STZ-exposed diabetic rats; in contrast, the boost of hippocampal CHOP expression in diabetic rats was reduced by means of NaHS (Figure 7). These findings have identified a function of suppression of ER stress by NaHS in hippocampus of STZ-exerted rats.

### NaHS reduces hippocampal cleaved caspase-12 expression in STZ-induced diabetic rats

**Figure 8:**
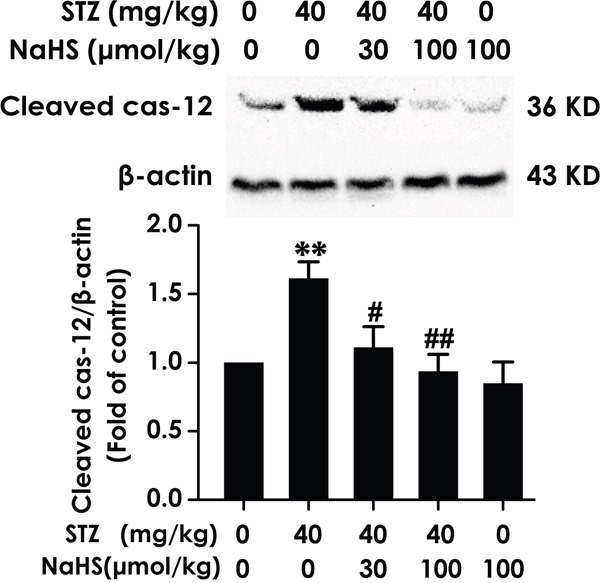
Effect of NaHS on the expression of cleaved caspase-12 in the hippocampus of STZ-induced diabetic rats Rats were once injected with STZ (40 mg/kg) and treated with NaHS (30 and 100 μmol/kg/d, ip) for 30 days. The expression of cleaved caspase-12 in the hippocampus of rats was detected by Western blot using an anti-cleaved caspase-12 antibody and β-actin was used as a loading control. Values are expressed as the mean ± S.E.M. (n=3-5). **P < 0.01, versus control group; ^#^*P* < 0.05, ^##^*P* < 0.01, versus STZ-treated alone group.

Cleaved caspase-12 is a crucial mediator of apoptosis induced by ER stress. Thus, we turned our attention to cleaved caspase-12. As schematized in Figure 8, hippocampal cleaved caspase-12 was significantly upregulated in STZ-induced diabetic rats; however, treatment with NaHS clearly suppressed the upregulation. This was further supported that a physiological role for NaHS treatment in protect against STZ-induced hippocampal ER stress.

### NaHS enhances the generation of hippocampal endogenous H_2_S in STZ-exposed diabetic rats

**Figure 9:**
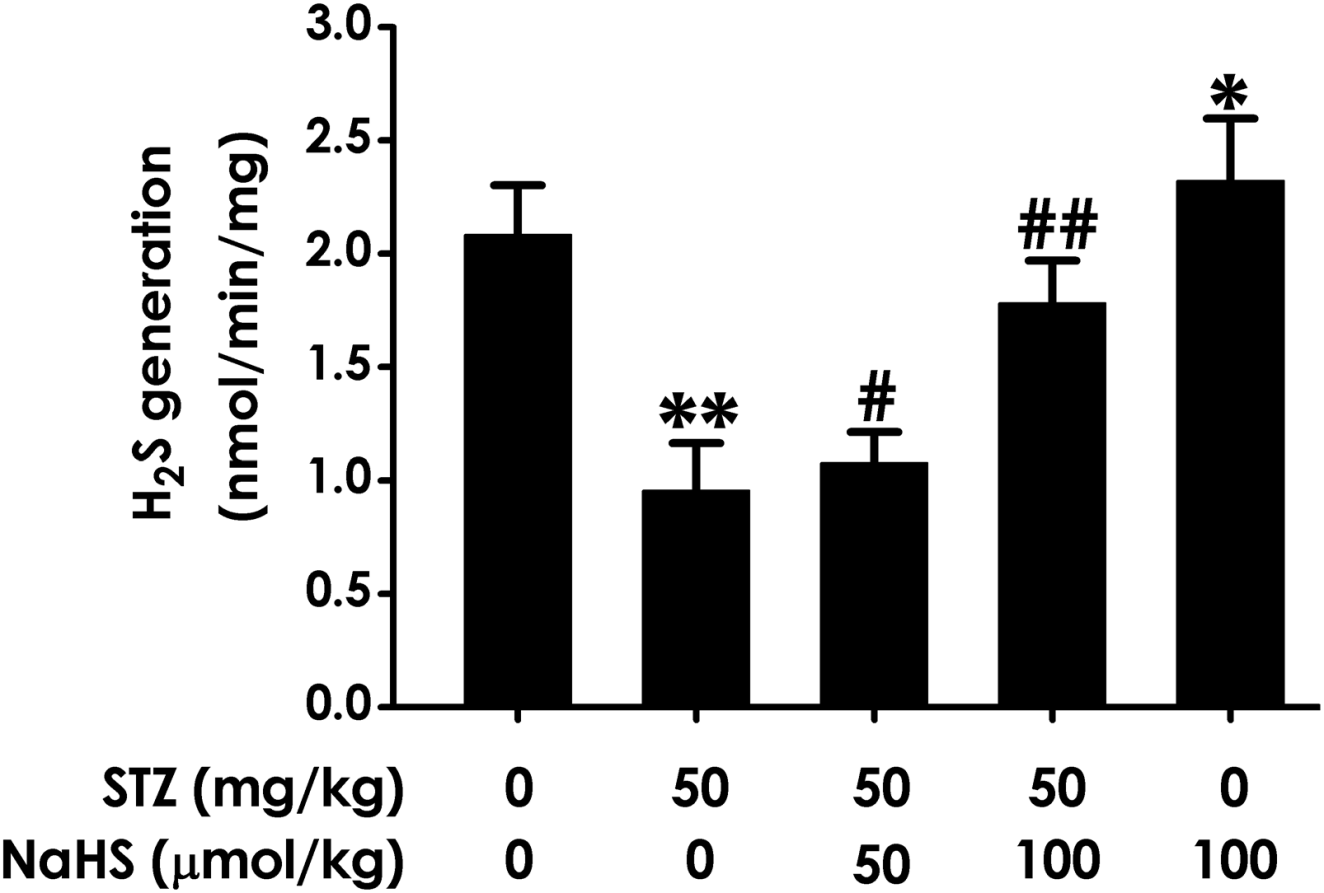
Effect of NaHS on the generation of hippocampal H2S in STZ-induced diabetic rats Rats were once injected with STZ (40 mg/kg) and treated with NaHS (30 and 100 μmol/kg/d, ip) for 30 days. The generation of hippocampal of rats was assayed by sensitive sulphur electrode. Values are expressed as the mean ± S.E.M. (n=3-5). *P< 0.05, *P < 0.01, versus control group; ^#^*P* < 0.05, ^##^*P* < 0.01, versus STZ-treated alone group.

To broaden our understanding of the improvement of cognitive impairment as well as the role of NaHS, we identifies whether the generation of hippocampal endogenous H_2_S in STZ-induced diabetic rats is decrease and whether NaHS reverse this decrease, we performed sensitive sulphur electrode to measure generation of hippocampal H_2_S. As schematized in Figure 9, hippocampal endogenous H_2_S generation in STZ-induced diabetic rats was significantly decreased compared to blank group. However, NaHS evidently prevented the decreased of hippocampal H_2_S generation in diabetic rats. These data show the important role played by NaHS in improve diabetic cognitive impairment induced by ER stress was rely on enhances the generation of hippocampal H_2_S.

## DISCUSSION

It has been demonstrated that the protective effect of H_2_S against the learning and memory decline in experimental AD [16, 17]. The metabolic disorder of diabetes is known to associated with the damage of central nervous system (CNS), which is often believed to contribute to changes in neurotransmission and structural malignant transformation, even interferences of learning and memory [33, 34]. In our experiments, we investigated the effects of H_2_S on the diabetes-associated cognitive impairment and the underlying mechanisms. We provided evidence that NaHS improves the cognitive impairments in STZ-exposed diabetic rats. In parallel to this, NaHS attenuates the hippocampal ER stress in STZ-exposed diabetic rats. NaHS is now regard as a H_2_S donor in the current research, which generates H_2_S through direct interaction of dissociated HS- and H+ in solution. [35, 36]. H_2_S is known as a toxic gas and its toxicity associated with concentration. H_2_S can be generated in the brain and the endogenous H_2_S under physiological conditions in the brain is a relatively high concentration at 50–160 μmol/L [37]. The concentration of H_2_S induced by NaHS in the present work is close to its physiological concentration. NaHS at 100 μmol/kg/d did not alter the cognitive function, which indicated NaHS in the present work has no toxicity to rats. Therefore, our results for the first revealed that H_2_S is an important protective factor against cognitive dysfunction in DM and the underlying mechanism is involved in prevention of ER stress in the hippocampus.

The MWM test has been identified as a mode to assess rodentine spatial learning and memory, which get wide spread acceptance [38]. In MWM, the spatial learning of rats is evaluated through the hidden-platform acquisition test and the spatial memory is investigated in the probe trial test. We showed that STZ-induced diabetic rats exhibited an increase in the escape latency in training session, and decreases in the crossing platform times and the percentage of time spent on the target quadrant when implemented probe trail, which indicated STZ-exposed diabetic rats have the descendant of spatial learning and memory. Of important, NaHS-treated diabetic rats showed that the escape latency and the journey in the goal quadrant were markedly decreased and that the duration spent on the goal quadrant as well as the time of crossings of the area of goal were markedly enhanced. Further, the visible platform test supported the conclusion by ruling out change in animal swimming ability and vision that may also influence spatial learning and memory. Thus, it was reasonable to believe that H_2_S could improve the spatial learning and memory performance in the diabetic rats.

In addition, we tested the working memory functions of rats in the transfer phase of the hidden platform version of the MWM. In this experiment, we transferred the platform to the contrary quadrant, and their capacity for learning and remembering the new platform location were measured. STZ-induced diabetic rats had difficulty in forming a good working memory of the new platform location, as evident by that they exhibited significant higher escape latency and a significant increase in the swim journey to find the hidden platform during 4-d training trials compared to control group rats and explored the new training quadrant and crossed the platform location significantly less often than did the control rats in a probe trial 1 day after the end of training. Taken together, results of the escape latency, travelled distance in the goal quadrant, duration spent on quadrant, and times of platform crossings indicated that H2S improves the working memory performance in STZ-induced diabetic rats. The Y-maze task is a specific and sensitive test for working memory in a new environment [39, 40]. In the Y-maze test, we found that administration of H_2_S led to a marked increasing in correct rate in diabetic rats. This result also indicates the potential role of H2S to improve the STZ-exposed diabetic working memory disorder.

The NOR test on the basis of the differential exploration of familiar and new objects [41], and it is used to study short-term, declarative memory and attention. In the present study, we showed that in NOR, rats treated with STZ displayed a decreased discrimination index, which also indicates the cognitive dysfunction. Furthermore, the protective action of H_2_S in diabetic cognitive dysfunction was further supported in novel object recognition test. Administration of H_2_S dose dependently upregulated the discrimination index of diabetic rats in the novel object recognition test.

ER stress is often believed to have a great importance in the induction of insulin resistance and diabetes [25, 42, 43]. The importance of the hippocampus for cognitive functions is well established [44, 45]. Especially, spatial memory strongly based on activity of hippocampus min rats is widely accepted [46]. When cells were exposed to stress or damage, ER stress is a early or initial cellular response, which is associated with neuronal death in many neurodegenerative diseases. [47]. ER stress-induced apoptosis in the hippocampus is assessed to clarify the mechanism of diabetic cognitive impairment [29]. Therefore, we speculated that H_2_S has function as efficient anti-cognitive disorder by alleviating hippocampal ER stress. Our experiments showed that the hippocampal expressions of GRP78, CHOP, and cleaved caspase-12 in diabetic rats were all increased. In consistent with the previous report [29], our data also suggested that hippocampal ER stress is often believed to contributes to the diabetic cognitive dysfunction. It is to be noted that administration of H_2_S significantly inhibited the expressions of above proteins in the hippocampus of STZ-induced diabetic rats. These results suggested that H_2_S have physiological role in downregulating the elevated ER stress in the hippocampus of diabetic rats. Our recent data also demonstrated that H_2_S protect PC12 cells from formaldehyde exposed ER stress [48]. These published findings was further explained and supported our present results. Thus, the present work revealed that H2S improves the diabetic cognitive dysfunction through inhibiting the hippocampal ER stress. It is interesting to note an opposite finding that H_2_S increases ER stress in Beta cells *in vitro* [49]. The difference in observed model, pancreatic beta cells *in vitro* and hippocampus tissues *in vivo*, may account for the discrepancy between the two studies. In order to further demonstrated that improving the diabetic cognitive impairment by suppressing ER stress indeed depend on the increase in hippocampal endogenous H_2_S, we investigated whether NaHS treatment changes the hippocampal endogenous H_2_S generation in STZ-induced diabetic rats. The hippocampal endogenous H_2_S generation of diabetic rats is decrease, while this downregulation is reversed by intraperitoneal NaHS. Therefore, we suggested that NaHS-provided protection in diabetic rats depend on the increase in the hippocampal endogenous H_2_S generation. However, it has been reports that H_2_S formation in pancreas and liver was markedly increased in STZ-induced diabetic rats [50]. This conflict is probably due to the discrepancy of organs.

In conclusion, our results clearly indicated that diabetes mellitus triggers neurocognitive decline in adult rats and H_2_S prevents this neurofunctional impairment as demonstrated by the MWM, the Y-maze, and the novel object recognition tests. We also showed that H_2_S attenuates the ER stress in the hippocampus of diabetic rats. These finding implied the inhibition of hippocampal ER stress is involved in H_2_S-enhanced the cognitive function in diabetic rats. Our findings suggested that H_2_S is a possible candidate for the prevention and treatment of cognitive dysfunction in diabetes mellitus. However, the further mechanisms of this protective action of H_2_S in diabetic cognitive impairment will be in depth explored in our future work. Furthermore, we will carry out this experiment in clinically relevant model progressively.

## MATERIALS AND METHODS

### Drugs

NaHS (dissolved in nonpyrogenic 0.9% NaCl and filtered through a 0.2 μm filter) was obtained from Sigma-Aldrich (St Louis Missouri, USA). Streptozotocin (STZ) was purchased from MP Biomedicals, LLc (Santa Ana, California, USA). Specific monoclonal anti-GRP78 and anti-CHOP antibodies were purchased from Epitomic Inc (Burlingame, UK). Specific monoclonal anti-cleaved caspase-12 antibody was obtained from Sigma Chemical (St Louis, MO, USA).

### Animals and treatments

**Figure 10:**
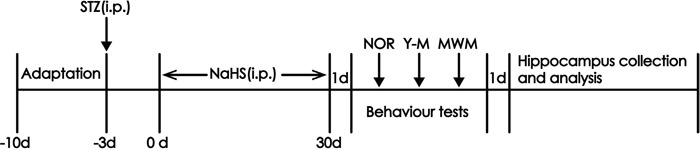
Schematic diagram of the experimental schedule NOR, novel objects recognition test; Y-M, Y-maze test; MWM, Morris water maze test.

Adult male Sprague-Dawley rats (280-300g), obtained from the SJA Lab Animal Center of Changsha (Changsha, China). Rats were given free access to food and water, and kept on a 12 h light/dark cycle (lights on 08:30–20:30) and under constant temperature (23 ± 1°C) and humidity (60%). After one week of acclimatization to the laboratory conditions, rats were randomly divided into five groups: Control group, received daily intraperitoneal (i.p.) infusions of normal saline; STZ-treated alone group, received a single intraperitoneal infusion of STZ (40 mg/kg) and 30-d infusions of normal saline (i.p.); the co-treated with STZ and 30 μmol/kg/d NaHS group, received a single intraperitoneal infusion of STZ (40 mg/kg) and 30-d infusion of NaHS (30 μmol/kg/d, ip); the co-treated with STZ and 100 μmol/kg/d NaHS group, received a single intraperitoneal infusion of STZ (40 mg/kg) and 30-d infusion of NaHS (100 μmol/kg/d, ip); and 100 μmol/kg/d NaHS (i.p.)-treated alone group, received an intraperitoneal infusion of NaHS (100 μmol/kg/d) for 30 d. After diabetic rat model was established, we injected NaHS. Behavioral tests that assess cognitive ability were performed 24 h after the last injection of NaHS. The experiments were always conducted between 9:00 and 17:00 h, which were carried out in accordance with the National Institutes of Health Guide for the Care and Use of Laboratory Animals and were approved by the Animal Use and Protection Committee of University of South China. All efforts were made to minimize the number of animals used and their suffering. Within one day after the behavioral tests, animals were killed and the hippocampus region tissues of the brain were rapidly removed on the ice-cold artificial cerebrospinal fluid and stored at −80°C for analysis (Figure 10).

### Experimental induction of diabetes

Diabetes was induced in rats by using an earlier reported method [51]. In brief, STZ was dissolved in 0.1 M sodium citrate buffer, pH 4.4 and administered at the dose of 40 mg/kg through i.p. route. STZ-treated rats received 5% of glucose solution instead of water for 24 h after injection of STZ in order to reduce death due to hypoglycemic shock. Blood samples were taken from the tail vein 72 h after STZ injection to measure blood glucose levels. Only animals with fasting blood glucose levels over 16.7 mmol/L were considered diabetic and used for the further study [2].

### Novel object recognition test

The novel object recognition test was performed according to the method described previously [52, 53], in order to assess the ability of rats to recognize a novel object in a familiar environment. We performed a novel object recognition test for 3 days including a habituation phase (10 min for one day), a training phase (5 min for one day) and a test phase (5 min for one day) in each rat. During the habituation phase, rats were placed into an open-field apparatus consisting of a rectangular area (50 cm wide × 50 cm long × 40 cm high) allowed to adapt for 10 min for each mouse without objects. Then, during the training phase, two identical objects were presented to each rat for 5 min. Twenty-four hours after the training phase, one of the old objects was replaced with a novel object and presented to each rat for 5 min during the test phase. Object exploration time was recorded using a video-assisted tracking system. The cognitive function of rats is measured by the discrimination index [= (novel object exploration time − familial object exploration time)/total exploration time × 100%]. To control the odor cues, the open field arena and the objects were thoroughly cleaned with alcohol, dried and ventilated for a few minutes between rats.

### Y- maze test

As described previously [52, 54], Y-maze (90 cm long × 90 cm wide × 76 cm high) was made of black-colored acryl and consists of three arms (A, B and C) at 120° angles to each other. Rats were habituated in the Y maze recording room for 30 min. During the habituation phase, rats were placed into the intersection of three arms and allowed to move freely 2 min. And then, rats were replaced in the intersection of three arms and allowed to move freely through the maze and to enter as many arms as they like during the 5-min sessions of test phase. Arm entry sessions were recorded when the hind paws of the rats were completely placed in the arm. Consecutive entry into three arms in an alternative order was defined as successive entries on overlapping triplet sets, and the correct response ratio was calculated as the ratio of overlapping triplet times/total entry times. The cognitive function of rats is measured by the correct response ratio.

### Morris water maze (MWM) test

Cognitive function of rats was assessed by using Morris water maze test as described earlier [55–57]. The experimental apparatus consisted of a circular water tank (180 cm in diameter, 60 cm in height), filled with water (23 ± 1°C) to a depth of 30 cm, which was rendered opaque by adding milk powder. The pool was divided virtually into four equal quadrants, labeled 1–2–3–4. A colorless escape platform (12.5 cm in diameter and 38 cm in height) was placed in one of the four maze quadrants (the target quadrant) and submerged 2.0 cm below the water surface during acquisition trails. The platform remained in the same quadrant during the entire experiment. The pool was located in a quiet test room, surrounded by many visual cues outside of the maze which was visible from within the pool and could be used by the rats for spatial orientation. Swimming was recorded using a camera capture, and analyzed using the MT-200 Morris image motion system (Chengdu Technology and Market Corp, Chengdu, China).

### Acquisition trail

During the place navigation training, each rat received four training periods per day with an intertrial interval of 60 s for four consecutive days. In each trial, the rat was gently placed into the pool at the middle of the circular edge in a randomly selected quadrant, with the nose pointing toward the wall and allowed a 120-s swim to find the platform. If rats failed to find the escape platform within 120 s by themselves, they were placed on the platform for 20 s by the experimenter and their escape latency was accepted as 120 s. After climbing onto the platform, the animal remained there for 20 s before the commencement of the next trial. The path and the latency to escape from the water maze (finding the submerged escape platform) were calculated for each trial.

### Probe trail

On day 5, the probe test was performed by removing platform and allowing each rat to swim freely for 120 s. The start position for each rat corresponded to one of two positions remote from the platform location in counterbalanced order. The time that rats spent in the target quadrant (where the platform was located during hidden platform training) and the number of times the rats crossed where the platform had been located were measured and calculated.

### Transfer acquisition trail

One day after completion of probe trail, each rat was test in the transfer phase of the hidden platform version. In this test, the platform was moved to the opposite quadrant and rats were received four trails per day with an intertrial interval of 60 s for four consecutive days to learn and remember the new platform location. The path and the latency to escape from the water maze (finding the submerged escape platform) were calculated for each trial.

### Transfer probe trial

One day after the end of transfer acquisition trail, the transfer probe test was performed by removing platform and allowing each rat to swim freely for 120 s. The time that rats spent in the target quadrant (where the platform was located during the transfer phase of hidden platform training) and the number of times the rats crossed where the platform had been located were measured and calculated.

### Visible platform test

After the transfer probe test, visual, motor, and motivation skills were also tested with a visible platform to rule out the possible deficits in sensorimotor processes. The platform was raised 2 cm above the water surface. The platform was moved to a novel quadrant in the pool at a fixed location for the four consisted trials. The latency to reach the platform and the average speed were recorded.

### Western blot analysis

The expression of GRP78, CHOP or cleaved caspase-12 was measured by Western blot. After sacrifice, the entire hippocampus was removed and homogenized in ice-cold homogenizing buffer (20 mM Tris-Cl, pH 7.4, 150 mM NaCl, 1 mM EDTA, 1% Triton X-100, 1 mM PMSF. After centrifugation at 12,000 for 30 min at 4°C, the supernatant was collected and the protein content was subsequently assayed by using a BCA Protein Assay Kit (Beyotime, Shanghai, China). Equal quantities of total protein (30 μg per lane) were separated by sodium dodecyl sulfate-polyacrylamide gel electrophoresis (SDS-PAGE), followed by transfer to nitrocellulose membranes. The membranes were incubated in5% milk at room temperature for 2 h. Blots were then incubated with primary antibodies including rabbit monoclonal antibody for CHOP (diluted 1:1000), GRP78 (diluted 1:2000), cleaved Caspase12 (diluted 1:1000), or β-actin (1:2000). After washing with buffer, the blots were incubated in anti-rabbit secondary antibody-conjugated with horseradish peroxide (1:5000) in TBS-T with 5% milk at 4°C overnight. The signal of the immunoblots was visualized using an image analysis system equipped with a software BIO-ID (Vilber Lourmat, France).

### Sensitive sulphur electrode

The generation of hippocampal endogenous H_2_S was assayed by using sensitive Sulphur electrode as described methods previously [58, 59]. Hippocampus region tissue (50 mg) was isolated from brain of anesthetized rats (n=3) and made 10% tissue homogenate in 50 mmol/L Phosphate Buffer (PH6.8). After centrifugation at 12,000 g for 30 min at 4°C, the supernatant was gathered. Cryovial test tubes (2 mL) were used as the center wells each containing 0.5mL of 1% zinc acetate as trapping solution and a filter paper of 2.0-2.5 cm^2^ to increase the air/liquid contacting surface. The reaction mixture contained 100 mmol/L Phosphate Buffer (PH7.4), 10 mmol/L L-cysteine, 2 mmol/L pyridoxal 5^’^-phosphate, and 10% (w/v) tissue homogenate and was added into the Erlenmeyer flask. The flasks containing reaction mixture and center wells were flushed with N2 before being sealed with a double layer of parafilm. Reaction was initiated by a thermostatic water bath for 90 min at 37°C and was stopped by addition of 0.5 mL of 20% trichloroacetic acid. The flasks were sealed again and incubated at 37°C for 60 min in the shaking water to ensure a complete trapping of the H_2_S released from the mixture. The content of H_2_S in the solution was measured by Sulphur ion-selective electrode (Unisense, Aarhus, Denmark) and the H_2_S production was expressed as unit nmol/min/mg protein.

### Statistical analysis

Data are expressed as mean ± S.E.M. The significance of inter-group differences was evaluated by two-way analyses of variance (ANOVA: Least-significant difference test). Statistical significance was considered at P< 0.05.
